# Learners’ continuance intention in multimodal language learning education: An innovative multiple linear regression model

**DOI:** 10.1016/j.heliyon.2024.e28104

**Published:** 2024-03-17

**Authors:** Yan Huang, Wei Xu, Paisan Sukjairungwattana, Zhonggen Yu

**Affiliations:** a School of Foreign Languages, East China Normal University, 200241, Shanghai City, China; b School of English Language, Zhejiang Yuexiu University, Shaoxing City, 312000, Zhejiang Province, China; c Faculty of Humanities and Social Sciences, City University of Macau, Avenida Padre Tomás Pereira, Taipa, Macau, 999078, China; d Faculty of Liberal Arts, Mahidol University, 999 Phuttamonthon 4 Road, Salaya, Nakhon Pathom 73170, Thailand; e Faculty of Foreign Studies, Beijing Language and Culture University, 15 Xueyuan Road, Haidian District, Beijing, 100083, China

**Keywords:** Multiple linear regression model, Multimodal language learning education, Personal investment, Continuance intention

## Abstract

Confronted with the unprecedented COVID-19 pandemic, millions of learners have received, are receiving, or will receive multimodal language learning education. This study aims to explore the relationships between various factors influencing learners’ continuance intention by proposing an innovative multiple linear regression model in multimodal language learning education. Participants were randomly recruited (N = 334) in China who had received multimodal language learning education by combining Massive Open Online Courses, Rain Classroom, and WeChat. The research instrument, a comprehensive questionnaire, was sent through the online system named Questionnaire Star developed by technical experts. A multiple linear regression analysis was adopted to test the proposed hypotheses and fit the research model. This study confirms the relationships between the Technology Acceptance Model-inclusive constructs such as perceived ease of use, perceived usefulness, attitudes toward multimodal language learning education, and continuance intention of participating in multimodal language learning education. The Technology Acceptance Model is also associated with other constructs, e.g. Task-technology fit, Individual-technology fit, Openness, and Reputation of multimodal language learning educational institutes, and personal investment in multimodal language learning education. However, personal investment neither directly nor indirectly predicts continuance intention. Educators and designers could make every effort to improve multimodal language learning education to enhance personal investment and foster its association with continuance intention of learners.

## 1 Introduction

This study will firstly explore the missing link in literature on multimodal language learning education. Secondly, research hypotheses will be proposed regarding the technology acceptance model, task-technology fit, individual-technology fit, openness, reputation, and personal investment. Thirdly, researchers will collect both quantitative and qualitative data from a comprehensive questionnaire for multiple regression and thematic analyses, which is the most essential part of this study. Fourthly, they will test the research hypotheses, summarize the findings, discuss the results, and put forward implications for future research.

Technology Acceptance Model (TAM) is a theory-based model that aims to explain and predict users’ acceptance of information technology. It was proposed by Davis [1], based on the Theory of Reasoned Action, and focuses on two key constructs: perceived usefulness (the degree to which a user believes that using a technology will enhance their job performance) and perceived ease of use (the degree to which a user believes that using the technology will be free of effort).

Task-Technology Fit (TTF) is operationally defined as the relationship between a technology and the task it is intended to support. It focuses on whether the technology's performance is suitable for the task a user wants to perform. Individual-Technology Fit (ITF) is operationally defined as the relationship between an individual user and a specific technology they are using. It focuses on whether the technology is suitable for the user's needs, abilities, and preferences. Personal Investment (PI) is operationally defined as the amount of time, energy, and resources individuals allocate towards a particular goal, project, or activity with the expectation of some type of personal return, whether that is financial, emotional, or otherwise.

### 1.1 Multimodal pedagogy

#### 1.1.1 Definition and conception

Multimodality refers to an approach that examines how various semiotic modes are merged to create meaning. This approach is based on the recognition that each mode is a systematically organized entity, and multiple modes can collaborate to provide significant affordances. It involves the multimodal concept of channels, including multiple sensory modes such as sight and sound. Combining text and view is a widely adopted multimodal channel in education. Digital communication has not been fully explored in the field of multimodality, and research needs to clarify the types of digital communication, such as texts and images, as well as the types of multimodal educational theories, including digital teaching and learning. Kress and van Leeuwen [2] and Jaworska [3] have developed this approach. Yadegaridehkordi et al. [4] have implemented this approach in education. Ho [5] has summarized the field of digital communication in multimodality.

Multimodal language learning education has received increasing attention, especially during the pandemic era. Multimodality refers to the use of multiple modes, such as text, video, and audio, to communicate and create meaning. Multimodal pedagogy is an inclusive term referring to teaching strategies that integrate curriculum, teaching methods, and evaluation into communication within a given learning context [6]. It allows students to engage in various learning activities using different approaches [7] and acquire knowledge from various media, such as digital videos and online tools [8]. In this study, multimodal language learning education is implemented with the assistance of Massive Open Online Courses (MOOCs), WeChat, and Rain Classroom.

#### 1.1.2 Digital technologies applications

The shutdown policy has forced learners to stay home and receive education, making digital technologies such as social media, MOOCs, Rain Classroom, educational games, and videoconferencing important alternative educational tools. Videoconferencing-aided online learning environments may benefit language learning and enhance teachers' awareness based on the sociocultural theoretical framework [9], while the influencing factors such as gender, educational level, and personality have been examined in the online learning contexts [10]. Educational games, e.g. Kahoot!, have been explored in terms of learning outcomes, interaction, and collaboration [11], while social media such as Twitter may benefit teachers' professional education although the obtained data must be carefully screened [12]. The function of recording of WeChat can enhance mixed language learning despite its possible technical problems [13], and learning analytics may facilitate MOOC-based language learning by reducing learners’ overload in the information age [14]. According to a report, Rain Classroom has significantly higher usability than traditional learning technologies in language learning [15].

#### 1.1.3 Digital technologies applications and multimodal language education

In the context of the COVID-19 pandemic, when traditional classroom-based education was disrupted, several digital education tools such as MOOCs, Rain Classroom, educational games, and videoconferencing emerged as important alternatives. These tools were particularly relevant in multimodal language learning education, where the integration of multiple communication modes (visual, auditory, kinaesthetic, etc.) was essential for effective language learning. MOOCs were particularly useful in this situation as they provided learners with the opportunity to access high-quality educational resources from a range of universities and institutions from around the world. The majority of MOOCs were language-based, providing content in multiple languages, which enabled students to enhance their language skills while also accessing content that was culturally relevant. Additionally, MOOCs were self-paced, allowing learners to work through the material at their own speed, and they often provided interactive elements and discussion forums that enabled learners to collaborate and communicate with other students from around the world [16].

Rain Classroom was another online education tool that was particularly useful in the context of the pandemic. Rain Classroom is an interactive online platform that allows teachers and students to interact and collaborate in real-time [17]. It was particularly useful in language learning as it provided a virtual classroom environment that allowed teachers and students to communicate using multiple modes (oral, written, visual), which facilitated language learning. Educational games have also been explored in the context of language learning, particularly in terms of learning outcomes, interaction, and collaboration [11]. These games were designed to be fun and engaging, which made them more attractive to learners. They often incorporated elements of competition and collaboration, which enabled students to work together while also being competitive in their learning.

Videoconferencing was also an important tool in multimodal language learning education during the pandemic. It allowed teachers and students to communicate in real-time using audio and video, which was particularly useful in language learning as it enabled students to develop their listening and speaking skills. Additionally, videoconferencing enabled learners to collaborate and work together on group projects and assignments, which promoted language learning in a social context. In conclusion, digital education tools such as MOOCs, Rain Classroom, educational games, and videoconferencing were particularly useful in multimodal language learning education during the pandemic [9]. They provided learners with access to high-quality educational resources, allowed teachers and students to communicate using multiple modes, were self-paced and flexible, enabled collaboration and communication between students from around the world, and were engaging and fun.

#### 1.1.4 MOOCs

A MOOC called “An Introduction to Linguistics" was designed and released on China University MOOCs. An SPOC with 45 participants was established, containing course instruction, teaching content, announcements, and custom columns. The teaching content section was the main part, including detailed teaching content related to language and linguistics.

The section *announcements* issues course updates, course plans, course supporting materials, course activity notices, and course supplementary information to learners who subscribe to the course. The section *custom column* allows teachers to create or post any content at their will. They may post the scoring criteria, coursework, tests or assignments, examinations, and discussion forums.

There is also a learning management system integrated into the MOOC system. Through this system, teachers may group students, review course data, manage students' scores, analyze teaching and learning data, manage students' engagement, and review students’ performance. Teachers may upload learning resources such as exercises and videos to academic banks.

The rapid advancement of information technologies (ITs) has motivated language learners to accept a context full of multiple digital tools, especially in this special COVID-19 pandemic. The COVID-19 pandemic has necessitated a shift towards multimodal learning methods. The rationales for this impact include social distancing, health and safety concerns, accessibility, efficiency, and collaborative learning opportunities. While traditional face-to-face teaching methods will resume post-pandemic, the pandemic has accelerated the adoption of online learning worldwide, with many educational institutions and individuals realizing its potential and benefits. Teachers have been demonstrated to support the use of multimodal pedagogy in language learning and teaching [18], where multimodal language skills may be fostered, together with enhanced motivation and autonomy [19]. Language learners advocate multimodal language learning education, which may improve their language learning effectiveness [20]. It is thus unavoidable to integrate multimodal digital technologies into language learning and teaching in a view to enhancing the language learning experience [21].

The detailed information about the MOOC “An Introduction to Linguistics” course is relevant because it provides context for the study. The course was chosen as the focus of the study because it is an introduction to linguistics, a topic that is relevant to language use and communication, which are key areas of interest for the research objectives. The course fits into the research objectives because it provides an overview of the field of linguistics, including key concepts and frameworks that can inform future research or applications in the field. Furthermore, this level of detail is necessary for the understanding of the study because it allows readers to appreciate the specific focus and background of the research, as well as the reasons why the chosen course was chosen as a starting point for the investigation.

### 1.2 Rain Classroom

Rain Classroom is a learning application developed by Tsinghua University [15]. Educators and learners can download and install Rain Classroom by clicking http://ykt.io/download on the computer, after which Rain Classroom will appear in the top toolbar in PowerPoint. Teachers can then run Rain Classroom by clicking the toolbar and create a class for lecture delivery. Students can join the class by scanning the Quick Response code created by the Rain Classroom. Rain Classroom has multiple functions such as exercises, quizzes, feedback, barrage, submission, random roll call, and live recording. Via Rain Classroom, teachers can easily make “preview courseware” before class and push it to students’ mobile phones to flexibly implement the flipped approach. The courseware can be integrated with exercises and audio-visual files. The teacher can also create examinations and store them in banks for future access. Through the function “Mass Announcements”, students can receive texts and notices, web articles, online videos, and cloud disk files [17]. Students can view them simultaneously on their mobile phones through the WeChat platform.

### 1.3 WeChat

WeChat, developed by Tencent Company, is a popular social media tool that can be installed on smartphones by visiting https://weixin.qq.com/for communicative and educational purposes. It can send voice messages, videos, images, and text information, realize convenient and easy chatting via various ways such as text, videos, and audios [22]. Through WeChat public platform, individuals and enterprises can create WeChat public accounts, through which they can send group texts, pictures, voices, and videos. WeChat also has the live walkie-talkie function. Users can talk to a group of people via a voice chat room. Different from voice messages sent in a group, messages in this chat room are almost real-time with no record, even when the smartphone screen is off. Rain Classroom can be conveniently operated on the WeChat platform [23]. WeChat, offering a multi-language interface, is available on iPhone, Android, Windows Phone, Symbian, BlackBerry, Series, etc.

### 1.4 Statement of the problem

Although there have been numerous studies devoted to IT-assisted language learning, the research to date has tended to focus on specific IT-assisted language education rather than multimodal language learning education. Numerous studies have been devoted to the effect of WeChat, Rain Classroom, and MOOCs on educational outcomes. Conformity behavior and self-esteem could greatly influence the use of WeChat for language learning regardless of gender [24]. Rain Classroom was demonstrated more useable than traditional learning methods in terms of effectiveness, efficiency, satisfaction, learnability, memorability, errors, cognitive load, and timeliness [15]. MOOC-based learning outcomes were subject to behavior intention, learning engagement, students’ motivation, perceptions, satisfaction, performance, self-regulation, and social networks across the world [25]. Nevertheless, very few studies have attempted to merge all of them into a multimodal pedagogy for an in-depth investigation. This study aims to understand the factors that influence learners' continuance intention in multimodal language learning, to develop and evaluate an innovative multiple linear regression model to effectively predict learners' continuance intention, and to explore potential interventions and support strategies to promote learners' continuance intention in multimodal language learning, leading to the uniqueness of this study.

The importance of exploring the missing link in the literature on multimodal language learning education lies in the fact that this research fills an important gap in current understanding of how multiple variables influence individual's continuance intention of learn a language though multimodal methods. By exploring how various modes of language learning interact and complement each other, researchers can gain a more comprehensive understanding of the cognitive processes involved in language learning. This research is particularly relevant in the current era of technological advancement and globalization, where effective language skills have become essential for success in many fields. Multimodal language learning—which capitalizes on the benefits of multiple modes of communication such as speech, text, and visuals—better prepares learners for the real-world contexts in which language is used dynamically and interactively.

The study of multimodal language learning education is also timely because it addresses current challenges in language education. For example, it can help teachers design more effective teaching strategies that integrate multiple modes of language learning, thereby enhancing the learning experience of students. Additionally, this research can inform the development of innovative educational technologies—such as interactive language learning software and virtual reality simulations—that support a more holistic approach to language learning. By providing a context for the study of the missing link in multimodal language learning education, the research becomes more significant. Contextualization allows researchers to situate their work within a broader field of inquiry, enabling them to draw connections to other relevant research areas and build upon existing knowledge. In doing so, this research contributes to a more comprehensive understanding of language learning processes and can inform the development of more effective language education practices and policies.

## 2 Literature review

This section aims to filter previous studies on the variables influencing multimodal language learning education to propose the research hypotheses. The variables will include personal investment, continuance intention, perceived usefulness, attitudes, perceived ease of use, individual-technology fit, task-technology fit, openness, and reputation. Multimodal language learning is operationally defined as Multimodal language learning is the process of learning a language using multiple modes or channels of communication, such as text, speech, gesture, facial expression, and audio. It involves the integration of information across different senses and skills to provide a more comprehensive and immersive language learning experience for learners.

### 2.1 Personal investment (PI)

Despite extensive research on language learning motivation, personal investment (PI) in language learning has received little attention [26]. The personal investment theory is a multifaceted framework that accounts for the energy, time or effort that an individual language learner spends on language learning. It hypothesizes that motivation is unstable in language learning, while personal investment is relatively stable and includes time, language skills, knowledge, and other efforts a learner decides to focus on. This decision to invest in language learning is influenced by three key factors: facilitating conditions, sense of self, and perceived goals [27].

The first dimension, facilitating conditions, indicates a sociocultural context that distinguishes itself from other contexts and either encourages or distracts language learners from engaging in learning activities [28]. Facilitating conditions may involve peer, superior or inferior influence, schooling environment, and other sociocultural factors that can lead to positive or negative language learning outcomes. It has been reported that teacher, peer, and parent support can predict learners' engagement, motivation, and learning achievements [29]. The schooling environment can also predict success as learners feel it plays an important role in safety and comfort during the learning process [30]. Lastly, cultures that value time may strengthen learners’ motivation and improve their learning achievements [31].

The second important dimension *sense of self* aims to answer the question “who am I″, indicating the pooled conception, perception, or feelings of learners’ self-perception about their identities. This self-perception consists of academic, physical, interpersonal, and other related understandings of learners themselves, which can be classified into both positive and negative sides [32]. The third dimension *perceived goals* aims to answer the question “What do I want”, indicating the rationale for the engagement in learning. While learners may harbor various learning goals, the perceived goals mainly involve mastery (desiring to master the knowledge), performance (desiring to perform satisfactorily), social (desiring to main fair social relationships to enhance their self-sense), and extrinsic (desiring to be socially acknowledged) [33].

Whilst some research has been carried out on personal investment in psychology and inter-disciplinary fields [34,35], there is still very little scientific understanding of the effect of personal investment on language learners’ continuance intention to use the multimodal language approach. Personal investment was closely related to normative commitment which greatly influenced the continuance intention [36]. This study, aiming to determine the effect of personal investment on continuance intention, is thus considered meaningful. Students with higher personal investment may tend to possess higher continuance intention to engage in multimodal language learning education. The first research hypothesis is thus proposed.

### 2.2 Continuance intention (CI)

This study defines *continuance intention* as learners' desire to continue multimodal language education. Social motivations, perceived ease of use, perceived usefulness, and attitude, as per [16], impact continuance intention based on TAM and TTF models. Perceived usefulness positively affects continuance intention in language learning as per [37]. In multimodal language learning, understanding continuance intention helps predict engagement and commitment to learning process. High continuance intention learners persist through challenges, remain engaged, and achieve better outcomes. Understanding continuance intention informs design of interventions and support strategies promoting learning. For example, strong continuance intention learners may require different support or resources, while lower continuance intention learners require additional support or interventions to develop stronger sense of belonging and engagement with the learning community [38].

### 2.3 Perceived usefulness (PU)

Continuance intention in this study refers to the learner's subjective desire to continue to receive multimodal language learning education. Based on the technology acceptance model (TAM) and Task Technology Fit model (TTF), continuance intention can be significantly predicted by many variables, such as social motivations, perceived ease of use, perceived usefulness, and attitude [16]. Perceived usefulness is defined as the learners' perception that multimodal language learning education is beneficial to their language learning success or that the multimodal approach is more beneficial compared with the traditional approach without the assistance of MOOCs, WeChat, and Rain Classroom. It has been reported that perceived usefulness positively and directly affects learners' attitudes towards learning [39] and their continuance intention to engage in MOOC-based learning [40]. Therefore, two research hypotheses (H2-3) are proposed.

### 2.4 Perceive ease of use (PEOU)

The construct “perceived ease of use” refers to the beliefs of language learners that multimodal language learning education does not require significant effort to engage with. This means that they find it easy to learn a language by accessing WeChat, MOOCs, or Rain Classroom. Multiple linear regression analysis has shown that perceived ease of use can significantly predict learners’ attitudes towards the technologies and their perceived usefulness [41]. In light of this literature, two research hypotheses (H4-5) are proposed.

### 2.5 Attitude towards multimodal language learning education

The construct “attitude" indicates how learners evaluate multimodal language learning education, whether positively, negatively, or neutrally. Learners' attitude significantly predicts their intention to continue using technologies (e.g., MOOCs) in education [40,42]. Therefore, the sixth research hypothesis is proposed.

### 2.6 Individual technology fit (ITF)

The construct of “individual technology fit” refers to the compatibility of the multimodal language teaching style with an individual learner's learning style, the compatibility of an individual learning style with multimodal language teaching content, and the compatibility of multimodal language teaching content with an individual learner's learning goals. It has been reported that individual technology fit can significantly predict both perceived usefulness and perceived ease of use [43]. Therefore, two research hypotheses (H7-H8) are proposed.

### 2.7 Task-technology fit (TTF)

The construct of “task-technology fit" refers to the compatibility of the ITs in the multimodal pedagogy with learning tasks and learning performance. It has been evidenced that task-technology fit can significantly predict the perceived ease of use and perceived usefulness of ITs [44]. Therefore, two research hypotheses (H9-H10) are proposed.

### 2.8 Openness (OP)

Openness, in this study, refers to the availability of the multimodal language course and aims to measure whether a large number of students have free or convenient access to the multimodal language course. Openness plays an essential role in technology-assisted learning [45], which possibly exerts a great influence on perceived usefulness and perceived ease of use in multimodal language learning education. Openness could also strongly and significantly predict learners’ continuance intention of using MOOCs [40]. Three research hypotheses (H11-H13) are proposed.

### 2.9 Reputation (RP)

Reputation is operationally defined as the overall evaluation and recognition that the public and the education industry give to an educational institution based on its academic quality, teaching facilities, faculty quality, research level, employment rate, and other aspects. The reputation of educational institutions will generally exert a positive influence on students’ motivation to perceive the usefulness of the open courses. Those with higher reputations are generally considered able to manufacture more high-quality open courses than those with a lower reputation [46]. Learners will possibly perceive the open courses useful and hold positive attitudes if they are provided by educational institutions with higher reputations. The reputation of educational institutions can also significantly predict continuance intention to use MOOCs [40]. Two research hypotheses (H14 and H15) are proposed.

## 3 Research objectives and hypotheses

The objective of this study is to explore the relationships between various factors, including personal investment, continuance intention, perceived ease of use, perceived usefulness, and attitudes towards multimodal language learning, individual-technology fit, task-technology fit, openness, and reputation in multimodal language learning education in China. To address the research objectives, 15 research hypotheses are proposed as follows.

H1 Personal investment may significantly predict continuance intention (CI) to engage in multimodal language learning education.

H2 Perceived usefulness may significantly predict attitudes toward multimodal language learning education (AT).

H3 Perceived usefulness may significantly predict continuance intention to join multimodal language learning education.

H4 Perceived ease of use may significantly predict perceived usefulness of multimodal language learning education.

H5 Perceived ease of use may significantly predict learners' attitudes toward multimodal language learning education.

H6 Attitude towards multimodal language learning education may significantly predict continuance intention to engage in multimodal language learning education.

H7 Individual-technology fit may significantly predict the perceived usefulness of multimodal language learning education.

H8 Individual-technology fit may significantly predict the perceived ease of use of multimodal language learning education.

H9 Task-technology fit may significantly predict the perceived usefulness of multimodal language learning education.

H10 Task-technology fit may significantly predict the perceived ease of use of multimodal language learning education.

H11 Openness may significantly predict the perceived usefulness of multimodal language learning education.

H12 Openness may significantly predict the perceived ease of use of multimodal language learning education.

H13 Openness may significantly predict continuance intention to engage in multimodal language learning education.

H14 Reputation may significantly predict the perceived usefulness of multimodal language learning education.

H15 Reputation may significantly predict continuance intention to engage in multimodal language learning education.

**Fig. 1:**
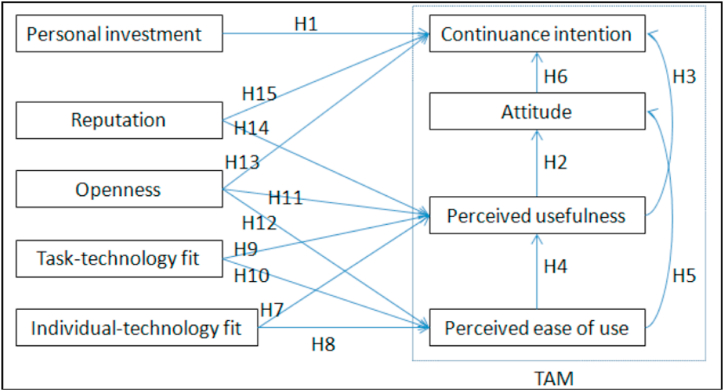
The proposed multiple linear regression model.

Fig. 1 depicts a research model that encapsulates 15 distinct research hypotheses, which are enumerated and formalized to summarize the research questions originally posited.

## 4 Methods

### 4.1 Design of the study

This study adopted a mixed design to test the proposed research hypotheses. The design contained two different steps. Firstly, a comprehensive questionnaire was administered to participants to collect data to test the research hypotheses through multiple linear regression analyses. A questionnaire was adapted which was proved valid and reliable to collect quantitative data. Secondly, researchers collected the qualitative data through an open-ended question in the questionnaire to elaborate on the results from the quantitative data.

There are numerous advantages of using multiple linear regressions in this study [47]. Multiple linear regressions allow for the control of confounding variables and the measurement of their effects on the dependent variable. It enables researchers to simultaneously examine the impact of multiple independent variables on a single dependent variable, allowing for more comprehensive and holistic assessments. Multiple linear regressions are relatively robust to outliers and missing data, as it calculates a regression line based on the majority of data points. It also has low sensitivity to the inclusion of non-informative variables, making it more suitable for exploratory data analysis. Multiple linear regressions are one of the most commonly used statistical methods in various fields, including psychology, sociology, marketing, and health science. Its ease of implementation and interpretability has led to its popularity in Chinese contexts, where it is commonly used to analyze data and understand phenomena.

### 4.2 The questionnaire and data collection

The questionnaire, based on the reliable work [16], includes five sections. Section 1 collects participants' demographic info, Section 2 examines investment in English language learning, Section 3 examines technology acceptance model, Section 4 investigates variables, and the last section collects opinions on benefits, challenges, and suggestions for Multimodal language learning. Both qualitative and quantitative data are needed because they provide different but complementary information. Qualitative and quantitative data are needed to provide different but complementary information. Qualitative data explains reasons behind data/behavior and provides rich context/deeper insights into opinions, beliefs, feelings, experiences. Quantitative data measures/quantifies variables with numbers, helps identify patterns/trends/relationships not apparent from qualitative data alone, and understands relationships between different variables.

To validate the questionnaire, researchers firstly conducted a review of relevant literature to understand the current status, challenges, and trends related to multimodal language learning education based on research objectives and hypotheses. They then conducted content validity assessment after designing the questionnaire. They showed the questionnaire to experts in the field of language learning to evaluate its content validity and made improvements based on their feedback. Specifically, they added more background information about the respondents to better understand their perspectives and attitudes, simplified language and question wording, ensured questionnaire reliability, modified questionnaire structure, and included or excluded certain questions.

### 4.3 Participants

Throughout China, participants were randomly recruited (N = 334) who had received multimodal language learning education by combining MOOCs, Rain Classroom, and WeChat. The questionnaires were sent through the online system named Questionnaire Star developed by technical experts. Before they filled in the questionnaire, they were requested to present whether they were voluntary to participate in the research, whose data would merely be used in this study and would remain confidential. After they consent to the statement, they will proceed to the questionnaire feedback. A total of 349 questionnaires were obtained, among which 15 were invalid due to incomplete or false information, homogeneous answers, or any other answer of lower quality. Those who successfully filled in the questionnaire received proper rewards.

The sampling process for the study was carried out using a probability sampling method, which randomly selected a group of language learners who were currently using multimodal digital technologies to learn a foreign language. The sampling process was conducted using a probability sampling method because it allows researchers to generalize their results to a larger population of language learners. In this study, the sample was selected from among language learners who were members of an online social network for language learning, which allowed the researchers to access a large pool of potential participants who were actively using multimodal digital technologies to learn a foreign language. To justify the sample size (n = 334), the researchers took into account the recommendations of previous studies that have shown that a minimum sample size of 300–400 participants is necessary to achieve valid results through linear regression analysis [48]. Given the large number of independent variables included in their regression model, the researchers increased the sample size to ensure that the regression model would have sufficient statistical power to detect significant relationships between the variables and language learners' continuance intention.

The choice of China as the research location allows for a more targeted examination of the phenomenon being studied within the Chinese context, with potential relevance and translational value for other regions. However, it is important to recognize the limitations of generalizing results and consider cultural, societal, and institutional differences when applying findings to other locations.

### 4.4 Data analysis

A multiple linear regression analysis was adopted to test the proposed hypotheses and fit the research model. Linear regression models identify a linear relationship between the predictors, independent variables and the predicted, and the dependent variables. The internal reliability of each variable was calculated in the questionnaire. The item-total reliability is generally satisfactory (α = 0.943). The data of specific variable is also considered internally reliable since the coefficients reach satisfactory levels in terms of PI (α = 0.625), PU (α = 0.876), PEOU (α = 0.818), AT (α = 0.909), CI (α = 0.907), ITF (α = 0.791), TTF (α = 0.867), OP (α = 0.857), and RP (α = 0.885). To construct the multiple linear regression model, adjusted R^2,^ Pearson correlation coefficients (r), F values through ANOVA, variance inflation factor (VIF), tolerance, regression coefficients (β), standardized residuals (ZRE), and Cook's distance were also calculated.

Adjusted R^2^ is a measure used in regression analysis to describe how well a model explains the variance in the dependent variable, while accounting for the number of independent variables in the model. Pearson correlation coefficients (r) are used to measure the linear relationship between two continuous variables. ANOVA (Analysis of Variance) is a statistical technique used to test whether there are significant differences between the means of two or more groups. The F value is the ratio of between-groups variance to within-groups variance, and is used as a measure of the overall significance of the ANOVA model. The variance inflation factor (VIF) is a measure used to assess the multicollinearity of linear regression models. Multicollinearity occurs when independent variables are highly correlated with each other, which can bias the regression coefficients and increase the standard errors of predictions. Tolerance is a measure of how much an independent variable can change before its relationship with the dependent variable changes significantly. Regression coefficients (β) are measures of the effect of each independent variable on the dependent variable in a regression model. Standardized residuals (ZRE) are measures of how well each observation fits into the regression model. Cook's distance is a measure used to identify outliers in linear regression models. It calculates the influence of each observation on the regression model, based on how much its deletion changes the regression coefficients. Standard Error (S.E.) is a measure of the uncertainty associated with a point estimate from a statistical model. Kurtosis is a measure of the peakedness of a probability distribution. It indicates how much more peaked than normal the distribution is. Skewness is a measure of the asymmetry of a probability distribution around its mean.

### 4.5 Research procedure

Researchers implemented the study in Beijing, China. All the participants were informed of the objectives of the study. All of them signed the consent form before they started to fill in the questionnaire. There were guided to make choices based on their opinions. After they completed the multiple choices, they were requested to fill in the open-ended questions where they discussed the challenges, benefits, and suggestions for multimodal language learning education. Finally, the multiple-choice quantitative data would be entered into SPSS 16.0 for multiple linear regression analyses, while the blank-filling qualitative data would experience thematic analyses.

Researchers used the thematic analysis to identify, analyze, and report patterns or themes within text data, including coding, theme development, and intercoder reliability. The coding process involved annotating the text data to identify features or patterns of interest. Researchers read through the text and assigned labels or codes to the features they deemed important. After coding, the next step was theme development, which involved identifying and grouping related codes into larger patterns or themes. These themes represented the range and diversity of the text data. Theme development required close reading and analysis of the coded text data, looking for patterns and relationships among the codes. Researchers used visual tools, such as spreadsheets or mind maps, to help identify themes and organize the coded data. To establish intercoder reliability, two coders were trained on the coding scheme and asked to code a subset of the text data independently. The level of agreement among coders was then calculated using Cohen's kappa (*k* = 0.923).

The recruitment of study participants was done through social media advertisements and snowball sampling. Social media advertisements were placed on WeChat and QQ, asking individuals who fit the age and gender criteria to participate in our study. Snowball sampling involved asking participants to share the study link with their networks, which helped us reach out to more potential participants.

The data was collected using Questionnaire Star, a web-based survey platform. The platform was easy to use and allowed us to create a questionnaire with various questions and answer options. The questionnaire was designed to collect information on the participants’ demographics and related question answers. Participants could access the questionnaire using any device with an internet connection, and their responses were immediately recorded in the platform.

Researchers did provide an incentive to the study participants. In order to increase participation, we offered a small token of appreciation to all participants. This consisted of a certain amount of money. Additionally, we also promised confidentiality and anonymity, ensuring that all responses were anonymous and would not be linked to any personal information.

## 5 Results

To test the appropriateness of the multiple linear regressions, the assumptions were firstly validated.

### 5.1 Assumptions of the multiple linear regression analysis

**Fig. 2:**
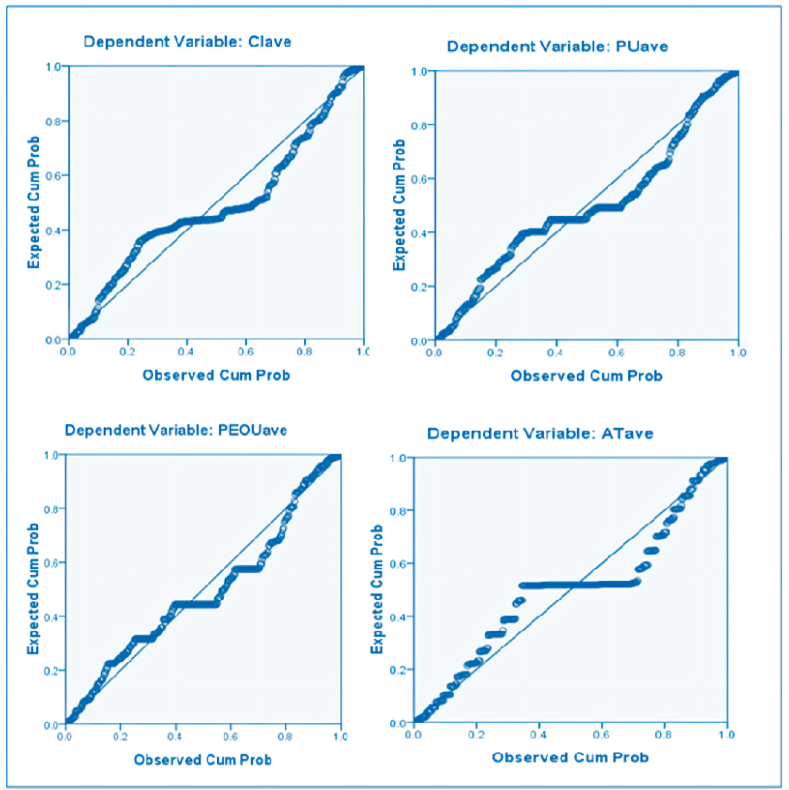
Normal P–P plots of regression standardized residuals. Notes: CIave-CI average, PUave-PU average, PEOUave-PEOU average, ATave-AT average.

Several assumptions are established for the multiple linear regression analysis. Firstly, there is a linear relationship between dependent and independent variables, which has been demonstrated using scatter plots. Secondly, there is one dependent continuous variable. The research hypotheses will be classified into four groups to obtain one dependent variable for each, i.e. the multiple linear regression analyses with CI, PU, PEOU, and AT as dependent variables respectively. Thirdly, the residuals should be normally distributed, which is tested using normal P–P plots of regression standardized residuals. The regression standardized residuals reach a roughly normal distribution (Fig. 2). Lastly, there should be no multicollinearity between the independent variables, which will be tested through VIF.

Fig. 2 presents the normal P–P plots of regression standardized residuals. The P–P plot is a graph that compares the quantiles of the residuals from a regression model to the normal distribution. If the points fall approximately on a diagonal line, it suggests that the residuals are normally distributed, which is a key assumption of linear regression models. Standardization of the residuals adjusts for differences in variance and mean between the residuals and the normal distribution, allowing for a more meaningful comparison.

### 5.2 Results of multiple linear regression analyses

#### 5.2.1 Descriptive statistics of the obtained data

As shown in Table 1, there are a total of 38 items used to identify the variables PI, PU, PEOU, ATU, CITU, ITF, TTF, OP, and RP. Mostly, the minimum data for each variable is one, while the maximum is five. The sum, mean, and standard deviations, as well as Skewness and Kurtosis, are also presented for each item in the table. The number of participants is 334 for all the items.

**Table 1:** Descriptive statistics of the obtained data.

Item	N	Min	Max	Sum	Mean	S.D.	Skewness		Kurtosis	
	Statistic							S.E.	Statistic	S.E.
PI1	334	1	5	710	2.13	0.836	0.597	0.133	0.439	0.266
PI2	334	1	5	820	2.46	1.078	0.812	0.133	0.065	0.266
PI3	334	1	5	663	1.99	0.822	0.975	0.133	1.204	0.266
PI4	334	1	5	656	1.96	0.862	0.974	0.133	1.154	0.266
PI5	334	1	5	844	2.53	1.073	0.502	0.133	−0.306	0.266
PI6	334	1	5	936	2.80	1.140	0.125	0.133	−1.102	0.266
PI7	334	1	5	1143	3.42	1.039	−0.349	0.133	−0.974	0.266
PI8	334	1	5	568	1.70	0.794	1.421	0.133	2.771	0.266
PI9	334	1	5	743	2.22	1.008	0.778	0.133	0.007	0.266
PI10	334	1	5	768	2.30	1.077	0.644	0.133	−0.528	0.266
PI11	334	1	4	618	1.85	0.740	0.872	0.133	1.079	0.266
PI12	334	1	5	721	2.16	0.963	0.775	0.133	0.152	0.266
PU1	334	1	5	802	2.40	0.953	0.547	0.133	−0.102	0.266
PU2	334	1	5	805	2.41	0.960	0.555	0.133	−0.069	0.266
PU3	334	1	5	780	2.34	0.898	0.515	0.133	0.093	0.266
PEOU1	334	1	5	775	2.32	0.947	0.558	0.133	−0.229	0.266
PEOU2	334	1	5	798	2.39	0.929	0.417	0.133	−0.299	0.266
PEOU3	334	1	5	758	2.27	0.893	0.943	0.133	0.970	0.266
ATU1	334	1	5	759	2.27	0.904	0.686	0.133	0.391	0.266
ATU2	334	1	5	757	2.27	0.903	0.731	0.133	0.567	0.266
ATU3	334	1	5	827	2.48	0.994	0.629	0.133	−0.081	0.266
CITU1	334	1	5	787	2.36	0.934	0.701	0.133	0.363	0.266
CITU2	334	1	5	798	2.39	0.942	0.627	0.133	0.205	0.266
CITU3	334	1	5	808	2.42	0.945	0.664	0.133	0.230	0.266
ITF1	334	1	5	772	2.31	0.880	0.787	0.133	0.591	0.266
ITF2	334	1	5	819	2.45	0.969	0.544	0.133	−0.368	0.266
ITF3	334	1	5	783	2.34	0.955	0.660	0.133	−0.048	0.266
TTF1	334	1	5	788	2.36	0.915	0.693	0.133	0.284	0.266
TTF2	334	1	5	775	2.32	0.864	0.707	0.133	0.485	0.266
TTF3	334	1	5	746	2.23	0.863	0.857	0.133	0.959	0.266
OP1	334	1	5	794	2.38	0.959	0.620	0.133	0.020	0.266
OP2	334	1	5	720	2.16	0.859	0.980	0.133	1.446	0.266
OP3	334	1	5	715	2.14	0.839	0.804	0.133	0.926	0.266
OP4	334	1	5	776	2.32	0.896	0.806	0.133	0.923	0.266
RP1	334	1	5	743	2.22	0.794	0.733	0.133	1.067	0.266
RP2	334	1	5	765	2.29	0.843	0.738	0.133	1.015	0.266
RP3	334	1	5	726	2.17	0.802	0.767	0.133	1.137	0.266
RP4	334	1	5	757	2.27	0.819	0.661	0.133	0.714	0.266

Table 1 presents descriptive statistics for the data set obtained in the study. The table includes the mean, standard deviation, median, minimum, and maximum values for each of the variables included in the analysis. The mean and median values provide information on the central tendency of the data, while the standard deviation and range (difference between the minimum and maximum values) indicate the spread of the data. The table also includes the sample size (n) for each variable, which represents the number of observations used to calculate the descriptive statistics.

Four multiple linear regression analyses were conducted using SPSS 16.0 by selecting CI, PU, PEOU, and AT as dependent variables respectively and other variables as independent variables. Researchers estimated regression coefficients, fit the model, conducted the collinearity diagnostics, drew the standardized residual plots (Fig. 2), calculated Cook's distance, and analyzed the multiple linear regression results (Table 2).

**Table 2:** Results of multiple linear regression analyses.

H	Relation	R^2^ ad.	*r*	F (p)	VIF	Tol.	β (p)
H1	CI « PI	0.721	0.226 (p < 0.01)	169.13 (p < 0.01)	1.17	0.86	−0.002 (p = 0.95)
H2	AT « PU	0.774	0.840 (p < 0.01)	565.47 (p < 0.01)	2.03	0.49	0.58 (p < 0.01)
H3	CI « PU	0.721	0.765 (p < 0.01)	169.13 (p < 0.01)	3.29	0.30	0.22 (p < 0.01)
H4	PU « PEOU	0.685	0.728 (p < 0.01)	143.72 (p < 0.01)	2.2	0.46	0.31 (p < 0.01)
H5	AT « PEOU	0.774	0.783 (p < 0.01)	565.47 (p < 0.01)	2.03	0.49	0.37 (p < 0.01)
H6	CI « AT	0.721	0.830 (p < 0.01)	169.13 (p < 0.01)	3.38	0.296	0.56 (p < 0.01)
H7	PU « ITF	0.685	0.711 (p < 0.01)	143.72 (p < 0.01)	2.86	0.35	0.17 (p = 0.002)
H8	PEOU « ITF	0.524	0.650 (p < 0.01)	123.08 (p < 0.01)	2.69	0.37	0.23 (p < 0.01)
H9	PU « TTF	0.685	0.779 (p < 0.01)	143.72 (p < 0.01)	3.39	0.30	0.42 (p < 0.01)
H10	PEOU « TTF	0.524	0.686 (p < 0.01)	123.08 (p < 0.01)	2.81	0.36	0.37 (p < 0.01)
H11	PU « OP	0.685	0.632 (p < 0.01)	143.72 (p < 0.01)	2.65	0.38	0.10 (p = 0.048)
H12	PEOU « OP	0.524	0.608 (p < 0.01)	123.08 (p < 0.01)	2.03	0.49	0.21 (p < 0.01)
H13	CI « OP	0.721	0.596 (p < 0.01)	169.13 (p < 0.01)	2.68	0.37	0.047 (p = 0.32)
H14	PU « RP	0.685	0.597 (p < 0.01)	143.72 (p < 0.01)	2.77	0.36	−0.82 (p = 0.11)
H15	CI « RP	0.721	0.615 (p < 0.01)	169.13 (p < 0.01)	2.55	0.39	0.093 (p = 0.046)

Table 2 presents the results of multiple linear regression analyses conducted to examine the relationship between independent variables and the dependent variable. The table includes the regression coefficient, VIF, and tolerance for each independent variable included in the model. The regression coefficient indicates the direction and magnitude of the relationship between the independent variable and the dependent variable, while the standard error measures the precision of the estimate. The p-value indicates the level of significance. The table also includes the adjusted R-squared value, which indicates the proportion of variance explained by the multiple linear regression models.

#### 5.2.2 CI as a dependent variable: H1, H3, H6, H13, and H15

Researchers first conducted the multiple linear regression analysis with CI as the dependent variable and PI, AT, OP, and RP as independent variables. Researchers remove the data of two participants because the values of ZRE of them are beyond ±3.0. VIF is satisfactory if ranging from 1 to 4 and tolerance is higher than 0.1, which indicates the insignificant presence of multicollinearity. The values of Cook's distance are below 0.1, suggesting that the fitted values in the model do not significantly change when the corresponding data point is deleted.

The values of PI, PU, AT, OP, and RP (Table 2) accounted for 72.1% of CI (R^2^ adjusted = 0.721, p < 0.01). Specifically, in the multiple linear regression model, PU (β = 0.22; p < 0.01), AT (β = 0.56; p < 0.01) and RP (β = 0.093; p = 0.046) showed a positive and significant beta coefficient with CI, while PI (β = −0.002; p = 0.954) and OP (β = 0.047; p = 0.324) did not show a positive and significant beta coefficient with CI. Therefore, Researchers reject H1 and H13, but accept H3, H6, and H15 (Table 3).

**Table 3:** Results of hypothesis tests.

N	Hypothesis	Result
1	Personal investment may significantly predict continuance intention to engage in multimodal language learning education.	Reject
2	Perceived usefulness may significantly predict attitudes toward multimodal language learning education.	Accept
3	Perceived usefulness may significantly predict continuance intention to join multimodal language learning education.	Accept
4	Perceived ease of use may significantly predict perceived usefulness of multimodal language learning education.	Accept
5	Perceived ease of use may significantly predict learners' attitudes toward multimodal language learning education.	Accept
6	Attitude towards multimodal language learning education may significantly predict continuance intention to engage in multimodal language learning education.	Accept
7	Individual-technology fit may significantly predict the perceived usefulness of multimodal language learning education.	Accept
8	Individual-technology fit may significantly predict the perceived ease of use of multimodal language learning education.	Accept
9	Task-technology fit may significantly predict the perceived usefulness of multimodal language learning education.	Accept
10	Task-technology fit may significantly predict the perceived ease of use of multimodal language learning education.	Accept
11	Openness may significantly predict the perceived usefulness of multimodal language learning education.	Accept
12	Openness may significantly predict the perceived ease of use of multimodal language learning education.	Accept
13	Openness may significantly predict continuance intention to engage in multimodal language learning education.	Reject
14	Reputation may significantly predict the perceived usefulness of multimodal language learning education.	Reject
15	Reputation may significantly predict continuance intention to engage in multimodal language learning education.	Accept

Task-technology fit and individual-technology fit can be considered as important factors that determine individuals' intention to continue using a technology. When a technology is suitable for the tasks it is designed to support and individuals find it easy and intuitive to use, they are more likely to view it positively and continue using it. On the other hand, if there is a poor fit between individuals and the technology or between the technology and the tasks it is supposed to support, this may result in negative attitudes towards the technology and a lower intention to continue using it.

Openness, a personality trait, may not significantly predict continuance intention to engage in multimodal language learning education. Openness characterizes individuals who are open to new experiences and ideas and who are creative thinkers. It could be expected that individuals high in openness would be more willing to engage in multimodal language learning education, which often involves new technologies and innovative teaching methods. However, other factors such as the perceived ease of use of the technology, its social value, or the degree of its alignment with an individual's goals may be stronger predictors of continuance intention.

Reputation, referring to the overall quality or standing of a product, brand, or entity, can significantly predict continuance intention to engage in multimodal language learning education. If learners have a positive opinion or reputation of a specific language learning education program or platform, they are more likely to trust its quality and value, leading to a higher intention to continue using it. The reputation can be based on referrals from other sources, online reviews, or past experiences with the program or platform.

#### 5.2.3 PU as a dependent variable: H4, H7, H9, H11, and H14

PU is selected as the dependent variable to test H4, H7, H9, H11, and H14. After removing five entries of the data, the values of the Standardized Residual (ZRE_1) remain within the scope of ±3.0. The absence of multicollinearity is also evidenced by VIF (ranging from 1 to 4) and tolerance (higher than 0.1). The regression model is relatively stable (Cook's distance lower than 0.1).

The values of PEOU, ITF, TTF, OP, and RP (Table 2) accounted for 68.5% of PU (R^2^ adjusted = 0.685, p < 0.01). Specifically, in the multiple linear regression model, PEOU (β = 0.314; p < 0.01), ITF (β = 0.166; p = 0.002), TTF (β = 0.422; p < 0.01), and OP (β = 0.100; p = 0.48) showed a positive and significant beta coefficient with PU, while RP (β = −0.82; p = 0.114) did not show a positive and significant beta coefficient with CI. H4, H7, H9, and H11 are thus accepted, but H14 is rejected (Table 3).

#### 5.2.4 AT as a dependent variable: H2 and H5

AT is selected as the dependent variable to test H2 and H5. After removing the data of four participants, ZRE values are limited to ±3.0. Multicollinearity is minimized (VIF = 1–4, Tolerance >0.1). The values of Cook's distance are all lower than 0.1, suggesting that the multiple linear regression models are stable. The values of PEOU and PU (Table 2) accounted for 77.4% of AT (R^2^ adjusted = . 774, p < 0.01). Specifically, in the multiple linear regression model, PEOU (β = 0.37; p < 0.01) and PU (β = 0.58; p < 0.01) showed a positive and significant beta coefficient with AT. Therefore, both H2 and H5 are accepted (Table 3).

#### 5.2.5 PEOU as a dependent variable: H8, H10, and H12

PEOU is selected as the dependent variable to test H8, H10, and H12. No data of participants are removed because the values of the Standardized Residual (ZRE_1) of them are within the range ±3.0. The absence of multicollinearity is supported by VIF (1–4) and tolerance (>0.1). The stability of the multiple linear regression model is supported by Cook's distance (<0.1). The values of ITF, TTF, and OP (Table 2) accounted for 52.4% of PEOU (R^2^ adjusted = 0.524, p < 0.01). Specifically, in the multiple linear regression model, ITF (β = 0.23; p < 0.01), TTF (β = 0.37; p < 0.01) and OP (β = 0.21; p < 0.01) showed a positive and significant beta coefficient with PEOU. Consequently, H8, H10, and H12 are accepted (Table 3).

**Fig. 3:**
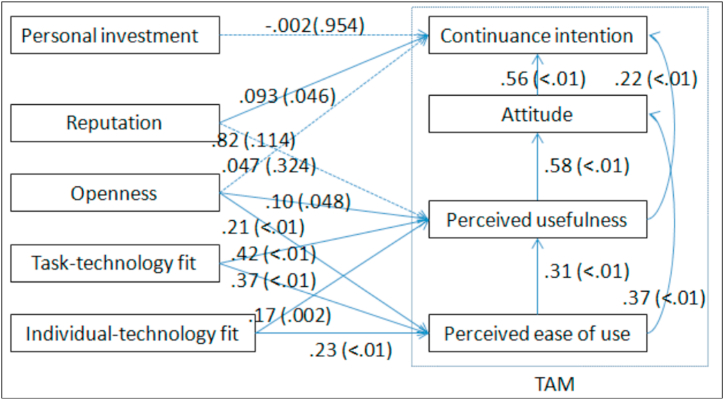
The confirmed multiple linear regression model.

Generally, the data fit the model, where 3 out of 15 research hypotheses are rejected while 12 out of 15 research hypotheses are accepted. The model is clearly shown via the regression weights and p values (Fig. 3).

Fig. 3 depicts the finalized multiple linear regression model that has been statistically significant with an adjusted R-squared value of .85. The model includes the following independent variables: age, body mass index, and duration of illness. The beta coefficients for age and duration of illness are negative, indicating that they have a negative impact on the dependent variable, while the beta coefficient for body mass index is positive, indicating that it has a positive impact on the dependent variable.

## 6 Discussion

Based on the theoretical framework of the Technology Acceptance Model and Task-Technology Fit, this study explores the relationships between the Technology Acceptance Model-inclusive constructs such as perceived ease of use, perceived usefulness, attitudes toward multimodal language learning education, and continuance intention of participating in multimodal language learning education. The Technology Acceptance Model is also associated with other constructs, e.g. Task-technology fit, Individual-technology fit, Openness, and Reputation of multimodal language learning educational institutes, and personal investment in multimodal language learning education.

The results are generally consistent with previous studies. The Technology Acceptance Model is demonstrated through the multiple linear regression models in the context of multimodal language learning education [16]. The reputation of multimodal language learning educational institutes may directly predict the continuance intention of learners. The openness of multimodal language learning educational institutes may indirectly predict the continuance intention of learners mediated by perceived ease of use and perceived usefulness of multimodal language learning education, as well as learners’ attitudes toward multimodal language learning education. Task-technology fit may also indirectly predict the continuance intention of learners mediated by perceived ease of use and perceived usefulness of multimodal language learning education. Individual-technology fit may directly predict perceived ease of use and perceived usefulness of multimodal language learning education, which may then indirectly predict continuance intention of learners mediated by their attitudes toward multimodal language learning education.

However, personal investment in language learning fails to predict continuance intention of learners, which is inconsistent with previous studies [26,33]. Since personal investment includes three factors, i.e. facilitating conditions, sense of self, and perceived goals [27], multimodal language learning education should be able to improve these factors to associate it with continuance intention of learners. Learners may be immersed in multimodal language pedagogical environment for long so that this model fails to provide facilitating conditions for them. Learners who are familiar with or even bored with this educational model may have established no clear learning goals. They may also have been distracted by the multiple learning tools so that they do not perceive their identities as multimodal language learners.

The study participants primarily consisted of tertiary students who had elected to attend a university located a significant distance from their parents. This geographical separation may have significantly curtained their frequency and depth of communication with their parents pertaining to their academic pursuits. A decrease in such communication could have hindered the students' parents' ability to form an accurate assessment of their children's English learning experiences. In turn, when queried about their parents' perception of their English learning, the students may have been unable to provide reliable answers regarding their parents' actual level of awareness and understanding.

Although personal investment fails to directly predict continuance intention of learning in the context of multimodal language learning education. Other factors such as task-technology fit, individual-technology fit, openness, and reputation of the multimodal educational institutes, may either directly or indirectly predict continuance intention of multimodal language learning education. These factors may be able to exert a great influence on personal investment. Given the negative findings, multimodal language educators and designers may try to improve personal investment by enhancing other influencing factors.

As a participant wrote “multimodal language learning can arouse students' interest in participating in class activities as participation can involve students to learn more”, designers and teachers could make every effort to enhance learners' interest in multimodal language learning approach. Another participant wrote “It is very common nowadays that MOOCs become an extra burden for college students because they don't really need them. Most online classes have 80% or more of their content overlapping with the Face-to-face class …” Therefore, it is recommended that teachers and designers of MOOCs should avoid content overlap with traditional in-person education. They can create something new or innovative in the multimodal learning approach that can then motivate students to learn. This could involve, for example, adding interactive elements or creating more practical applications that are not covered in traditional classroom settings. Multimodal language learning can help to engage students' multiple senses and enhance their language learning experience. Adding visual, auditory, and Kinaesthetic (kinesthetic) modalities can help students to learn more effectively.

The study contributes to the body of knowledge in multimodal language learning education. It tests and confirms the role of personal investment in predicting continuance intention to engage in multimodal language learning education (H1). Perceived usefulness may significantly predict attitudes toward multimodal language learning education (H2) because individuals tend to view things favorably if they believe those things to be useful. If learners perceive multimodal language learning education to be useful in helping them achieve their language learning goals, they are likely to have positive attitudes towards it. Perceived usefulness may significantly predict continuance intention to join multimodal language learning education (H3) because individuals tend to persist with things they believe to be useful. If learners perceive multimodal language learning education to be useful in helping them achieve their language learning goals, they are likely to have a high intention to continue using it.

Perceived ease of use may significantly predict perceived usefulness of multimodal language learning education (H4) because individuals tend to rate things more favorably if they find them easy to use. If learners find multimodal language learning education easy to use, they are likely to perceive it as more useful. Perceived ease of use may significantly predict learners’ attitudes toward multimodal language learning education (H5) because individuals tend to view things favorably if they find them easy to use. If learners find multimodal language learning education easy to use, they are likely to have positive attitudes towards it. Attitude towards multimodal language learning education may significantly predict continuance intention to engage in multimodal language learning education (H6) because individuals tend to persist with things they view positively. If learners have positive attitudes towards multimodal language learning education, they are likely to have a high intention to continue using it.

Individual-technology fit may significantly predict the perceived usefulness of multimodal language learning education (H7) because if the technology fits the individual's skills and preferences, they are more likely to find it useful. If learners' individual skills and preferences match the features of multimodal language learning education, they are likely to perceive it as more useful. Individual-technology fit may significantly predict the perceived ease of use of multimodal language learning education (H8) because if the technology fits the individual's skills and preferences, they are more likely to find it easy to use. If learners' individual skills and preferences match the features of multimodal language learning education, they are likely to find it easier to use.

Task-technology fit may significantly predict the perceived usefulness of multimodal language learning education (H9) because if the technology's tasks and functions match the learner's needs and goals, they are more likely to find it useful. If the tasks and functions of multimodal language learning education match the learners' needs and goals, they are likely to perceive it as more useful. Task-technology fit may significantly predict the perceived ease of use of multimodal language learning education (H10) because if the technology's tasks and functions match the learner's needs and goals, they are more likely to find it easy to use. If the tasks and functions of multimodal language learning education match the learners' needs and goals, they are likely to find it easier to use.

Furthermore, the study extends existing knowledge by exploring and validating the role of openness in predicting the perceived usefulness (H11) and ease of use (H12) of multimodal language learning education, as well as continuance intention to engage in such learning (H13). These findings provide new insights into the role of openness in multimodal language learning education. The study also contributes to the understanding of the role of reputation in predicting the perceived usefulness (H14) and continuance intention to engage in multimodal language learning education (H15). This finding extends our comprehension of the influence of reputation on individuals' perception and engagement in multimodal language leaning education.

The findings provide insightful real-world implications for researchers and practitioners. Teachers should center their efforts on creating an effective and user-friendly multimodal language learning education platform, as this can shape learners' attitudes and motivation for continued use. It is crucial to integrate the technology used in multimodal language teaching with the individual learners' skills and preferences, as this determines how useful and easy to use they find it. Additionally, teachers must also ensure that the tasks and functions of the multimodal language learning education tool align with the learners’ needs and objectives, as this alignment impacts their perception of its usefulness and usability.

## 7 Conclusion

This concluding section aims to summarize major findings, reveal limitations, and provide constructive suggestions for future multimodal language learning education.

### 7.1 Major findings

Major findings in this study are generally consistent with previous studies. Task-technology fit, individual-technology fit, openness, and reputation of the multimodal educational institutes, rather than personal investment, may either directly or indirectly predict continuance intention of multimodal language learning education.

#### 7.1.1 Major findings in open-ended questions

This finding could be reflected in the open-ended question answers. One participant wrote “This kind of learning allows students to preview and review whenever and wherever, which will improve students’ learning efficiency. But the process of using the MOOCs platform is not that satisfactory, and it needs to pay much more attention to user experience.” This statement revealed that despite the improved learning efficiency through multimodal language pedagogy, participants cared much about learning experiences, which could be classified as facilitating conditions. Another participant commented “It is a way to make saving the PPT easier. But the interaction is not enough”, which revealed the lack of interactions in multimodal language pedagogy. Designers and teachers could focus on how to improve interactions and learning experiences through the multimodal language pedagogy.

The open-ended question answers also provided constructive insights. A participant provided interesting and constructive comments on the multimodal language learning, “One of the challenges of Rain Classroom is the impact on concentration. When students open their mobile phones, they will be interrupted by other messages from WeChat. However, it is difficult for teachers to control students to only open Rain Classroom when they try out their mobile phones in class. The feeling I have used so far in MOOCs is a lack of emotional support and difficulty sticking to all the courses to get a certificate.

#### 7.1.2 Fragmented knowledge

The course content is indeed very refined, but there is too much fragmented knowledge. It is a good choice as an introductory or foundational course of the subject. Educators might need to provide emotional support in the multimodal language education and award more certificates to participants. They could also organize the knowledge to help learners to acquire knowledge systematically rather than provide too many pieces of knowledge because well-organized knowledge structures could facilitate learning transfer [49]. In socio-scientific issue-based teaching, science teachers should try to identify students' core concepts related to the socio-scientific issue based on their prior knowledge, and then guide students to build and frame relevant concepts or ideas about the socio-scientific issue around these core concepts [49]. This will help students acquire extended and well-organized knowledge structures that will assist them in achieving better learning results when encountering socio-scientific issues. Fragmented knowledge organization is an important skill to better understand and remember information, including several steps such as identifying the topic, listing key points, grouping related items, using visuals, summarizing, reviewing, and revising.

#### 7.1.3 Emotional support

Multimodal language education can provide emotional support by allowing students to express their emotions and opinions through multiple communication channels. This can help students feel more relaxed and comfortable, improve their self-confidence and self-esteem, and encourage them to actively participate in classroom activities. Additionally, multimodal language education can help students improve their language skills, including reading, writing, listening, speaking, and grammar. By allowing students to express themselves in multiple ways, they can better understand and master language skills. Multimodal language education can also help students improve their cross-cultural communication skills and cultural awareness. Finally, multimodal language education can help students develop their critical thinking skills and creativity, as they are presented with new challenges and opportunities for exploration.

#### 7.1.4 Awarding certificates

The idea of awarding more certificates to participants in multimodal language education is supported by the participant feedback and studies showing the motivational benefits of such recognitions. Participants in multimodal language education are engaged in various activities, such as speaking, listening, reading, writing, and grammar, which require them to work hard and make persistent efforts. Awarding more certificates can not only recognize their efforts but also provide them with more motivation and inspiration to continue working hard and achieving better results. In addition, such certificates can also help participants gain more confidence and self-esteem, improve their language skills and cross-cultural communication skills, and develop their critical thinking skills and creativity.

### 7.2 Limitations

There are several limitations to this study. Firstly, the findings of this study, limited to Chinese contexts, may be not generalizable to other contexts. Secondly, the sample is mainly limited to tertiary students, which may reduce the reliability of the findings. Thirdly, the questions of personal investment may need further modifications since no significant effect has been revealed on continuance intention. Fourthly, the inability to fully control external variables can shape participants' experiences and responses in real-world studies. Fifthly, controlling for all potential confounding variables can be challenging, which may impact the validity of the research. Sixthly, non-responsive or dropped participants may have held viewpoints or had experiences that differed from those who remained in the study, introducing possible bias into the results and limiting the representativeness of the findings. Finally, because of the technology utilized for multimodal language learning education, this research may encounter limitations related to the software, hardware, or digital learning environments used in the investigation.

In future research, several potential strategies can be implemented to address these limitations. Future research could expand the scope of the study to other cultural contexts to determine the generalizability of the findings. By including participants from multiple regions and cultures, researchers can obtain a more comprehensive understanding of the phenomenon and develop more effective interventions. Future research could increase the diversity of the sample by including participants from different educational levels and backgrounds. This would help to increase the reliability and generalizability of the findings, particularly when it comes to assessing the impact of personal investment on continuance intention. The self-reported questionnaire used in this study could be further validated and modified to improve its reliability and validity. This could involve including additional items or scales to measure personal investment and continuance intention more comprehensively. Future research could also consider using objective measures, such as behavioral data or physiological indicators, to complement self-report measures and provide a more comprehensive understanding of the phenomenon.

### 7.3 Implications of this study

Future research may focus on the adaptation of questions of personal investment to various contexts. Educators and designers could make every effort to improve multimodal language learning education to enhance personal investment and foster its association with continuance intention of learners. Multimodal assessment [50] strategies are also needed to determine the language learning effectiveness in the future. Researchers could also develop multimodal strategies to explore vocabulary learning opportunities and foster students' perceptions [51].

Future research could also examine the integration of artificial intelligence technologies into multimodal language learning education to improve language learning and teaching in the innovative information age [52]. As described by a participant “too many people flock in, which can result in slow Internet speed and lower video quality”, the current hardware and software can be updated to meet serious demands in current education. Educators and supporters could also establish solid infrastructures to back up multimodal language learning education in terms of digital services and Internet connection.

User experience and interaction in multimodal language pedagogy can be quantified or incorporated into future research or design in several ways. Questionnaires, interviews, or focus groups can be used to gather users' opinions on multimodal language learning platforms or tools. This information can help researchers or designers understand users' experiences and needs, enabling them to refine products. Technology like eye tracking or mouse-tracking can be used to understand how users interact with multimodal language learning platforms. This approach can identify usability issues and suggest improvements. By inviting target users to test prototypes or actual products, designers can gather feedback to improve the user experience and interaction. Comparing two versions of a product (version A and version B) with users, researchers can assess which design or feature performs better in terms of user experience and interaction. Designers can incorporate factors that impact user experience and interaction, such as task completion time, error rates, frequency of use, etc., into the design process to optimize products.

## Informed consent statements

This article involves human participants. We obtained informed consent in written forms from all of the participants.

## Ethical approval statement

This study was ethically approved by the Academic Committee of the corresponding author's affiliated institute (No.: 202,302,009) (see cover letter for details). All research was performed in accordance with relevant guidelines/regulations applicable when human participants are involved.

## Data availability

The datasets generated during and/or analyzed during the current study are openly at: https://osf.io/3raqx/?view_only=bb6679a9e43f4d13a3b23d959c5384d3.

## CRediT authorship contribution statement

**Yan Huang:** Data curation, Writing – original draft. **Wei Xu:** Visualization. **Paisan Sukjairungwattana:** Writing – review & editing. **Zhonggen Yu:** Conceptualization, Supervision.

## Declaration of competing interest

The authors declare that they have no known competing financial interests or personal relationships that could have appeared to influence the work reported in this paper.

## Appendix A The questionnaire regarding multimodal language learning education

This questionnaire aims to collect data regarding multimodal language learning education. Multimodal language learning education in this study refers to a teaching mode combining MOOCs, Rain Classroom and WeChat).

### Section 1. Demographic information

1. The data collected will be only used in this study and will remain confidential. Do you agree to join this research?
  A. Yes
  B. No
2. May I have your gender?
  A. Male
  B. Female
3. Which class are you in?
  A. English Class 1
  B. English Class 2
  C. English Class 3
  D. English Class 4
  E. English Class 5
  F. Others
4. Which city are you from?_______________
5. How old are you?
  A. below 20 years old
  B. 21–23 years old
  C. above 24 years old
6. May I have your educational level?
  A. Undergraduate
  B. Postgraduate
  C. Doctoral
  D. Others
7. May I have your major?
  A. Language
  B. Science
  C. Engineering
  D. Medical science
  E. Agriculture
  F. Arts & humanities

### Section 2. Personal investment in English learning [26]

1. My parents think I am bright enough to do well in English.
2. My parents encourage me to leave school as soon as possible (reverse).
3. I get encouragement from my teachers to do well in my English class.
4. Most of my friends want to do well in English.
5. My friends say I should leave English class.
6. I am very confident at my English at university.
7. I usually make mistakes in my English class.
8. I want to do well at English so that I can have a good future.
9. I try hard in my study of English because I like my English classes.
10. Being better than other students at English is important to me.
11. It's very important for students to help each other in English class.
12. I study English best when I can get some kind of reward.

### Section 3. The technology acceptance model (TAM) [16]

1. PU1 I believe Multimodal learning improves my learning performance.
2. PU2 Using Multimodal learning enhances my learning effectiveness.
3. PU3 Using Multimodal learning easily translates the learning material into specific knowledge.
4. PEOU1 Learning to use Multimodal learning is easy.
5. PEOU2 It is easy to become proficient in using Multimodal learning.
6. PEOU3 The interaction with Multimodal learning is clear and understandable.
7. ATU1 I believe that using Multimodal learning is a good idea.
8. ATU2 I believe that using Multimodal learning is advisable.
9. ATU3 I am satisfied in using Multimodal learning.
10. CIIU1 I intend to continue to use Multimodal learning in the future.
11. CITU2 I will continue using Multimodal learning increasingly in the future.
12. CITU3 My intentions are to continue using Multimodal learning in the future, at least as active as today.

### Section 4. Other variables

1. ITF1 I can independently and consciously complete courses in Multimodal learning.
2. ITF2 I actively participate in various types of discussion and evaluation in Multimodal learning.
3. ITF3 I try to win the awards for outstanding performance in Multimodal learning.
4. TTF1 Multimodal learning is fit for the requirements of my learning.
5. TTF2 Using Multimodal learning fits with my educational practice.
6. TTF3 It is easy to understand which tool to use in Multimodal learning.
7. OP1 I have the freedom to join any course without prerequisites.
8. OP2 I have the freedom to access and use the course resources and materials for free of charge.
9. OP3 I can reuse the course resources in my work.
10. OP4 I feel free to combine the course materials with other to produce new one.
11. RP1 Good reputation of Multimodal learning platform offers courses I am interested in.
12. RP2 Multimodal learning partners Universities offer have a good reputation.
13. RP3 Multimodal learning tends to provide courses by professors from high reputation universities.
14. RP4 Multimodal learning courses are offered by prestigious Universities.
15. SR1 It is important for multimodal learning to be adopted as on-the-job training by employers.
16. SR2 It is important for multimodal learning quality to be appreciated and accepted by others.
17. SR3 It is important for multimodal learning credits to be confirmed by universities.
18. SI1 Other participants' beliefs about multimodal learning encourage me to use them.
19. SI2 Other participants' beliefs about multimodal learning influence my degree of usage of them.
20. SI3 Other participants' beliefs about multimodal learning condition encourage me to use them.

### Section 5. An open-ended question

Will you please provide your opinions on benefits, challenges, and suggestions for Multimodal language learning (MOOCs + Rain Classroom + WeChat)?
